# Identifying risk of deliberate self-harm through longitudinal monitoring of psychological distress in an inpatient psychiatric population

**DOI:** 10.1186/s12888-015-0464-3

**Published:** 2015-04-14

**Authors:** Shraddha Kashyap, Geoffrey R Hooke, Andrew C Page

**Affiliations:** School of Psychology, The University of Western Australia, 35 Stirling Highway, Crawley, 6009 Western Australia; Perth Clinic, 21 Havelock Street, West Perth, WA 600 Australia; University of Western Australia & Perth Clinic, Perth, Western Australia

**Keywords:** Deliberate self-harm, Risk management, Suicidal ideation, Longitudinal measures

## Abstract

**Background:**

While cross-sectional correlates of deliberate self-harm, such as psychological distress, have been identified; it is still difficult to predict which individuals experiencing distress will engage in deliberate self-harm, and when this may occur. Therefore, this study aimed to explore the ability of longitudinal measurements of psychological distress to predict deliberate self-harm in a psychiatric population.

**Method:**

Participants (N = 933; age range 14–93 (*M* = 38.95, *SD* = 14.64; 70% female) were monitored daily in terms of suicidal ideation, depression, anxiety, worthlessness and perceptions of not coping. Latent Growth Curve Analysis was used to check if groups of inpatients reporting suicidal ideation, who shared early change in measures of psychological distress, existed. Logistic regression tested whether different groups were at higher (or lower) risks of deliberate self-harm.

**Results:**

Four groups were found. Of these, Non-Responders (high symptoms, remaining high) were more likely to engage in deliberate self-harm than patients with high, medium and low symptoms which improved over one week. Group membership was a greater predictor of deliberate self-harm than initial distress scores. Females and patients with personality disorders were significantly more likely to be Non-Responders.

**Conclusions:**

Continuous monitoring and subsequent grouping of inpatients according to their early change in psychological distress provides a novel and practical approach to risk management. A lack of early improvement in psychological distress may indicate a higher risk of deliberate self-harm.

## Background

Deliberate Self-Harm (including both suicidal behaviours and non-suicidal deliberate self-harm) is hard to predict and this makes it a difficult area of clinical case management. Non-suicidal deliberate self-harm refers to deliberate, self-inflicted harm on body tissue; not socially/culturally sanctioned and without the intent to die [1,2]. Suicide attempts refer to deliberate, self-inflicted, non-lethal injuries, with the intent to die [1]. Although non-suicidal deliberate self-harm differs from suicidal attempts in terms of the intent to die [3,4]; non-suicidal deliberate self-harm either separately or combined with previous suicide attempts can significantly increase the risk of future suicidal behaviour [5-7]. For example, individuals with multiple previous incidents of deliberate self-harm, a history of psychiatric admissions, substance abuse [5,8], and those who engaged in more severe cutting and burning [9] can be at risk of progressing to further suicidal behaviours.

Theoretical frameworks have been proposed to explain the link between non-suicidal deliberate self-harm, suicide attempts and future suicidal behaviour. For example, the Interpersonal Theory of suicide posits that while perceived burdensomeness and thwarted belongingness can lead to suicidal ideation as a first step, individuals need to acquire the capacity to harm themselves to act on those suicidal thoughts [10]. This capacity to harm oneself can be acquired either through non-suicidal deliberate self-harm, previous suicidal behaviour, or both forms of deliberate self-harm [2]. For example, the progression from less lethal deliberate self-harm to more lethal deliberate self-harm might occur through the habituation to physical pain [11,12]. This view has been supported by findings where previous non-suicidal deliberate self-harm was a strong predictor of future suicidal behaviour [4,13]. For example, non-suicidal deliberate self-harm was found to predict suicidal behaviour after controlling for depression [9,13,14], previous suicidal behaviour [13], hopelessness and symptoms of borderline personality disorder [14]. Finally, a prospective study found that non-suicidal deliberate self-harm in adolescents remained a significant predictor of future suicidal behaviour after accounting for depression and previous suicidality [15]. Therefore, exploring predictors of non-suicidal deliberate self-harm among people at risk of suicidal behaviour (e.g., those experiencing suicidal ideation) may help predict and prevent suicidal behaviour. That is, if a first step towards suicidal behaviour is to have thoughts about suicide, and the next step is to acquire the capacity (such as through non-suicidal deliberate self-harm); examining factors associated with non-suicidal deliberate self-harm amongst individuals who *already* report suicidal ideation might add to the precision with which future suicidal behaviour can be predicted. Indeed, since *both* non-suicidal deliberate self-harm and suicidal behaviour can increase the risk of future suicidal behaviour (e.g. [15]), both forms of self-injury are referred to as deliberate self-harm for the purposes of this study.

However, one difficulty with prediction may be the focus on taking cross-sectional measurements of potential risk factors of deliberate self-harm, such as psychiatric disorders and psychological distress [16], and expecting them to predict levels of a behaviour which might change over time. For example, a systematic review suggested that while most correlates of deliberate self-harm such as indicators of psychological distress have been recognized retrospectively, there is a lack of knowledge around proximal predictors, which require longitudinal studies to be identified [17]. It has also been argued that further research is needed to identify causal links between risk factors and deliberate self-harm [18]. For example, it is widely known that depression is associated with suicidal ideation, but it is difficult to predict which people with depression who are considering deliberate self-harm will actually engage in deliberate self-harm [18].

The difficulties in prediction may arise because factors influencing the risk of deliberate self-harm vary both within and between days [19]. Therefore, it is hardly surprising that a measurement taken at a single time point may struggle to predict the probability of an outcome, where its likelihood of occurring may fluctuate along with levels of risk factors. For example, items associated with deliberate self-harm such as suicidal ideation [19-21] can change depending on different situations or the presence of certain triggers [19,21]. Indeed, it was found that suicidal ideation in adolescents with Borderline Personality Disorder did not remain stable over 6 months [22]. Therefore, cross sectional measurements may not provide a valid measure of the variability in thoughts or feelings associated with deliberate self-harm at different times [19]. Therefore, it is still difficult to predict who will display deliberate self-harm or when, and with what consequence with sufficient precision to address this problem effectively.

A similar problem exists in psychotherapy research, where scores at the beginning of treatment provided imperfect prediction of post treatment outcomes and provided little information about individual responses to treatment [23]. Recognition of this difficulty led to “patient-focussed” research which suggested that individuals respond to treatment at different rates [24]. Importantly, knowing that people who improved rapidly in the early stages of treatment tended to have a better prognosis [25,26]; allowed researchers to identify the characteristics that distinguished the “early responders” from later responders (or those who deteriorate). Further it was found that groups of people shared distinct patterns of change, and that *early improvement* resulted in better treatment outcomes [24]. These results suggest that individuals respond to psychotherapy in different ways and that some individuals can be grouped according to shared early treatment responses. It may then be possible to determine who will *not* respond well to treatment by measuring their changes (e.g. in measures of psychological distress; [27]) during the early stages of treatment, and estimate their outcomes (e.g. deliberate self-harm) based on identified patterns [28]. For example, it is possible that *a lack of* early change in psychological distress may be associated with higher risks of engagement in deliberate self-harm. Continuously measuring change in psychological distress would then point to individuals who do not make early improvements.

Indeed, previous research showed that when suicidal ideation was monitored daily in an inpatient psychiatric hospital, where day 1 was the first day that inpatients reported suicidal ideation; five sub-groups of individuals were found who changed in their reported levels of suicidal ideation over 7 days at different rates [29]. It was also found that these sub-groups were associated with different levels of risk of engaging in deliberate self-harm, where the group who began with the highest levels of suicidal ideation and did not exhibit any early improvement was at the highest risk [29]. Therefore, to build on those results by studying the effects of other factors associated with both suicidal ideation and deliberate self-harm [6,12,15,27]; the existence of sub-groups who change at different rates on a *combination* of indicators of psychological distress, over 7 days of treatment were explored. These factors included; suicidal ideation [20], depression, anxiety [30-32], feelings of worthlessness [33,34] and perceptions of not coping [35-38]. A combination of distress factors were also found to be associated with an even higher risk of deliberate self-harm than one factor alone [34,39]. Therefore, by continuously monitoring *combined* measures of psychological distress during treatment; any groups of individuals who share early change on those factors can be identified. Risk of deliberate self-harm could then be estimated based on group membership. This estimation could be more precise than using cross-sectional measures of risk factors of deliberate self-harm alone; due to the potential for these factors to fluctuate over time.

In summary, psychotherapy research has shown that individuals can be grouped according to their shared patterns of early change in measures of psychological distress, where early improvements are associated with better outcomes [24,28]. Potential risk factors of deliberate self-harm, such as indicators of psychological distress may fluctuate and can be monitored daily. If individuals can be grouped according to shared early change in psychological distress during treatment, then certain groups may be at higher risks of deliberate self-harm, such as those who do not show early improvement. Identifying if these groups exist, and measuring the rates of deliberate self-harm in each group may improve the precision with which risk is estimated.

In addition, if these groups exist, and one group *is* at a higher risk of engaging in deliberate self-harm, characteristics which predict group membership should be explored. To this end, it was found that lower self-reported improvements in symptoms during treatment, along with higher symptom severity and younger age at admission to hospital were associated with higher rates of re-admission to hospital in a private inpatient psychiatric facility [40]. Higher rates of re-admission to hospital were also associated with greater problems with deliberate self-harm as assessed by clinical staff [40]. Therefore, number of admissions to this hospital was explored as a predictor of group membership. Furthermore, while the rate of deliberate self-harm in the adult general population is estimated to be between 4-6% and 20% in adult inpatient populations; rates were estimated to be higher during adolescence [2,37], and were found to approach 40% in adolescent inpatient populations [2]. Age was therefore explored as another potential predictor of group membership.

Furthermore, in a sample of 89 adolescents exhibiting recent deliberate self-harm in a psychiatric facility; 87.6% were found to fit diagnostic criteria for at least one psychiatric diagnosis [41]. Indeed, 67.3% of females met criteria for Axis II disorders where Borderline Personality Disorder (BPD) was the most common [41]. It was also found that adolescents exhibiting more and severe BPD symptoms were more likely to engage in deliberate self-harm [42]. Consequently, in the current sample; in addition to demographic variables such as gender, diagnostic categories may prove a useful avenue for exploration of predictors of group membership.

Therefore, this study aims to build upon previous research [29], to check if different groups of inpatients exist who change in their reported overall psychological distress during treatment at different rates. It then aims to explore whether different groups are at higher/lower risks of engaging in deliberate self-harm. Finally, it aims to check if demographic variables such as age and gender; the number of previous admissions to a private psychiatric hospital and diagnoses can predict group membership.

## Method

### Participants

The relevant measures were made available to inpatients at a 100 bed private psychiatric hospital which specialises in acute mental health care for both day-patients and in-patients, including Psychiatry, Clinical Psychology, Occupational Therapy and Nursing care. All inpatients were invited to complete measures, excluding those who chose not to participate, those that were being admitted/discharged on any particular day of measurement, patients who were on leave, patients not attending treatment, patients who had not yet been allocated a treatment group, and if clinical staff decided it was inappropriate due to factors such as cognitive impairment (e.g. patients undergoing Electro Convulsive Therapy). Further, patients were only chosen if they had a minimum length of stay of seven days, in order to examine changes in distress over several consecutive days. They were then selected if they completed the measure on a minimum of *three* occasions over seven *consecutive* days during their current admission (which is the required number of responses for conducting the longitudinal analyses [43]).

The total number of inpatients at the hospital during the time period 1st January 2011 to 13th March 2013 was N = 4258. Of these, N = 2538 (59.6%) completed the relevant measures. This study did not require any follow up measures.

Written informed consent and appropriate levels of consent from all patients was obtained, and the research was approved by the Human Research Ethics Office at the University of Western Australia.

### Final selection criteria

The base rate of deliberate self-harm amongst participants (N = 2538) was 4.3%. This population was then divided into those who never reported suicidal ideation during their admission (N = 1063, *rate of deliberate self-harm = 0.6%)* and those who did report suicidal ideation at least once during their admission (N = 1475, *rate of deliberate self-harm = 7.1%).* Patients who never reported suicidal ideation were excluded from the final sample. This is because this study was interested in rates of deliberate self-harm amongst people who do report suicidal thoughts during treatment, where deliberate self-harm occurring after reported suicidal ideation may indicate an acquired capacity for future suicidal behaviours [10].

This study was also interested in examining how patients expressing suicidal ideation changed in their psychological distress over time. To examine the time-course of changes in distress, it was important to ensure that the first time all patients expressed suicidal ideation was matched. To this end, as in previous research [29], scores for suicidal ideation were aligned with day 1 becoming the first day any patient reported thoughts about suicide.

Of the 1475 individuals who endorsed suicidal ideation, 542 did not complete the measures on at least three occasions. The final sub- sample of participants therefore included 933 voluntary inpatients at a private inpatient psychiatric clinic. Each patient was diagnosed by their treating psychiatrist, and the main primary diagnoses domains using the ICD-10 classifications [44] were Mood Disorders (55.1%), Neurotic, Stress-Related and Somatoform Disorders (18.4%) and Substance Abuse Disorders (9.8%). Cross-sectional measures were also used from this sample to predict deliberate self-harm using logistic regression [45].

### Outcome measures

#### Continuous and cross-sectional predictors of deliberate self-harms

Clinical change was measured by the Five Item Daily Symptom Index (DI-5; [27] a self-report symptom index developed to track patients’ perception of psychological distress daily during therapy. Patients were asked to complete the DI-5 Index daily as part of routine hospital data collection, and de-identified data were made available to researchers. The severity and frequency of symptoms were rated by patients on a six-point Likert scale, using the format; “Over the previous 24 hours I have felt [depressed]” with responses ranging from 0 (“at no time”) to 5 (“all of the time”). Items scores were added together and higher scores indicated more perceived psychological distress [27]. The DI-5 measures five separate items including depression, anxiety, worthlessness, not coping and suicidal ideation. This measure was found to be appropriate for use with a psychiatric sample as it correlated well with existing mental health measures such as the SF-36 Mental Health (r = −0.69, *p* < 0.01) and depression (DASS Depression; r = 0.65, *p* < 0.01) [27]. It also exhibited high internal consistency (Cronbach’s α = 0.88) and good test re-test reliability (r = 0.75) in a clinical sample [27]; as well as high internal consistency (Cronbach’s α = 0.82) and test re-test reliability (r = 0.72, p < .01) in the current sample. Finally, in the current sample, total symptom scores on day 1 correlated significantly with total DASS-Depression scores at admission (r = 0.48, p < .01). This study used the sum of scores for the 5 items (anxiety, depression, suicidal ideation, worthlessness and perceptions of not coping) on each day (DI-5 Index), for seven consecutive days as an independent and continuous variable.

The addition of scores into one variable (DI-5 Index) was deemed appropriate as confirmatory factor analyses (CFA) found that a one factor model provided good fit to the data in a clinical population [27]. Similarly, in the current sample, criteria described by [46] were used to check if a one factor model adequately fit the data in a CFA. The indices and criteria examined were; standardised root mean square (SRMR; good fit indicated by values close to 0.08 or below), the root mean square error of approximation (RMSEA; good fit indicated by values close to 0.06 or below); and the Tucker-Lewis Index (TLI) and Comparative Fit Index (CFI) which should be close to or more than 0.95 [46]. The CFI (0.98), TLI (0.96) and SRMR (0.03) indicated that a one factor model provided good fit to the data [46]. While the RMSEA (0.08) was close to indicating good fit, modification indices suggested that *anxiety* and *not coping* were correlated. After these terms were correlated, the RMSEA became 0.02 suggesting that fit improved absolutely. Overall, the weight of evidence points towards a one factor model providing adequate fit to the data.

The total score on the DI-5 for *day 1* for each patient in the sample was used as a cross-sectional measure, to compare predictive abilities on deliberate self-harm with the use of continuous measures over seven consecutive days.

#### Deliberate self-harm

Deliberate self-harm incidents were recorded by hospital staff on the risk management database. The information recorded is part of a standard recording of “risk events” by all Australian hospitals and includes a description of the incident, date and time it occurred and any actions taken. Incidents were categorised as non-suicidal deliberate self-harm (1), suicide attempt (2) and suicide (3), and actions taken were requiring no intervention/minor intervention/ medical assessment/enhanced level of observation; transfer to medical facility or discharged early. For the purpose of this study, only the *first* incidence of deliberate self-harm for each patient during the current admission was examined, and only the presence or absence of deliberate self-harm was studied (this included non-suicidal self-injury and suicide attempts).

#### Predictors of group membership

Age, gender, number of previous admissions and diagnoses were explored as potential predictors of group membership. This information was collected as part of normal hospital procedures and was available to authors.

#### Procedure

Patients were invited to complete the DI-5 on a touch screen every day from admission until discharge. Data included pre-treatment and treatment measurements of the DI-5 items for inpatients over seven consecutive days, during their first 30 days of admission; where day 1 was the first day that patients reported suicidal ideation.

### Statistics

This study first asked; can cross-sectional measurements such as the DI-5 Index on day 1 be used to predict rates of deliberate self-harm in an inpatient psychiatric population? Logistic regression was run deliberate self-harm (yes/no) as the dependent variable and DI-5 scores on day 1 as the independent variable [45].

The study then asked; do distinct sub-groups of individuals exist who share patterns of early change on the DI-5 Index over seven days; are different groups at different risks of exhibiting deliberate self-harm; and do variables such as age, gender, diagnoses predict group membership? To answer these questions, a Latent Growth Curve Analysis (LGCA; [47]) was run using the *Mplus* software [48] to check for groups of inpatients who change in their psychological distress at different rates. The validity of groups found using the LGCA were tested using chi square analyses, which measured any significant differences between groups and rates of deliberate self-harm [45]. Effect sizes were calculated using the Phi statistic, which measured the strength of association between two categorical variables [45]. This was followed by logistic regression analyses to check for any significant associations of age, gender, or diagnoses with group membership [45].

#### Data analysis

To deal with missing data full information maximum likelihood (FIML) was used [49]. Little’s MCAR tests were non-significant on the DI-5 Index, suggesting that data was missing at random and that no systematic patterns of missing data were present which could confound results. LGCA analyses were then run using a total index variable, where scores for each item were added together on each of the seven time points (days 1–7).

To obtain the best fitting LGCA solution the following indices were examined [50-52]. These included the Bayesian Information Criteria (BIC; [53]) which measures the goodness of fit and parsimony of the model, where a lower BIC indicates better fit [52]. In addition, the Vuong-Lo-Mendell-Rubin Likelihood Ratio Test (LMR-LRT; [54]) and the Parametric Bootstrapped Likelihood Ratio Test (BLRT; [52]) check whether the change in values for models with increasing number of classes is significant [24]. Further, high posterior probabilities (i.e. probability for most likely latent class membership; [50] high entropy (a measure of the quality of classification of individuals into latent classes; [24]) and higher log-likelihood values were also taken into account when choosing the optimal number of latent classes. Finally, based on the recommendations of [52]; the number of classes being explored stopped increasing the first time the LMR-LRT became non-significant. Further, [24] argue that there is substantial data demonstrating that there is a negatively accelerated (or log-linear) relationship between the amount of treatment provided and progress during treatment. Therefore, log-linear latent growth curve models were tested (see Table 1).

**Table 1:** **LGCA Model Fit Indices for the DI-5 Index (N = 933)**

**Number of classes** ***Log-Linear***	**2**	**3**	**4**	**5**
Log-likelihood value	−11675.66	−11384.43	−11262.65	−11212.06
Adj. BIC	23395.27	22823.81	22591.23	22581.72
Entropy	.88	.84	.81	.79
Posterior probabilities	.96, .97	.95, .93, .90	.88, .91, .93, .86	.78, .92, .90, .86, .83
LMR-LRT	*p<.01*	*p<.01*	*p<.01*	p =.20
BLRT	*p<.01*	*p<.01*	*p<.01*	*p<.01*

## Results

The 53 incidences of deliberate self-harm for individuals in the sample (N = 933) consisted of the following; 73.6% cutting or scratching, 7.5% punching surfaces, 5.7% burning, two self-reported attempted suicides (3.8%), and other instances of deliberate self-harm (9.4%). Due to the small number of reported suicide attempts (2 out of 53 incidents), and that this study aimed to predict risk of deliberate self-harm based on previously reported suicidal ideation and severity of distress; all forms of self-injury are referred to as deliberate self-harm and no distinction was made between suicidal and non-suicidal deliberate self-harm.

It was also found that five individuals engaged in their first incidence of deliberate self-harm *before* reporting suicidal ideation. These included two incidents of burning, two incidents of superficial cutting and one incident of punching a surface. Their mean age was 31.2 years old (SD = 14.6), and all 5 individuals were female. Since this study aimed to estimate risk of deliberate self-harm in the presence of suicidal ideation and based on early change in distress during treatment; those individuals were excluded from analyses predicting deliberate self-harm. This is because any self-harm occurring *before* an expression of suicidal thoughts was beyond the scope of this study to predict. However, they were not excluded from the LGCA investigating any sub-grouping according to early change in reported distress, as the first aim of this study was to check if those groups existed in the sub-sample of patients reporting suicidal ideation.

**Figure 1:**
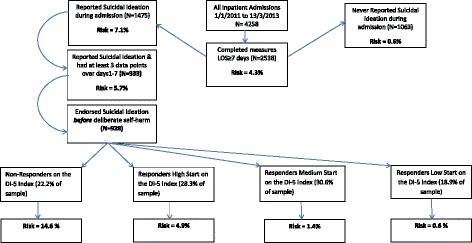
Comparing risk of deliberate self-harm between inpatients admitted 1^st^ January 2011 to 13^th^ March 2013.

Levels of missing DI-5 responses from participants from days 1–7 were as follows; 0%, 38.4%, 48.1%, 51.2%, 52.6%, 54.1%, 39.5%. Of the sub- sample, 653 were female (70%) and ages ranged from 14 to 93 years old (*M* = 38.95, *SD* = 14.64). The rate of deliberate self-harm in this sample (N = 933) consisting *only* of inpatients who reported suicidal ideation and fit selection criteria was 5.7% (see Figure 1).

*Part 1: predicting deliberate self-harm using a cross-sectional measure*

The predictive value of a cross-sectional measure (initial distress; DI-5 day 1) on deliberate self-harm in the final sample (N = 928), was compared with the predictive value of the DI-5 groups (days 1 to 7). Higher DI-5 scores on day 1 were found to have a weak positive relationship with deliberate self-harm (*Exp. B (1.2)*, *p < .01;* B = *.15*(SE = .04), Nagelkerke *R*^*2*^ *=* .06). Therefore, the next step was to check if monitoring symptoms and grouping patients according to their rates of change increased power in influencing odds of deliberate self-harm.

*Part 2: latent growth curve analyses*

Table 1 presents the model fit indices for the 2, 3, 4 and 5 DI-5 Index log-linear solutions. The 4 class log-linear solution was chosen as the optimal solution after considering all indices which indicated that it was the most reliable.

**Figure 2:**
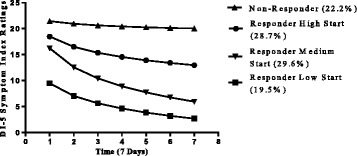
LGCA showing four trajectories of change for DI-5 Index over 7 days (N=933).

Figure 2 shows that individuals could be grouped according to their shared early responses to treatment over seven consecutive days in this clinical population, on the DI-5 Index. These groups were; Responder Low Start Class (19.5%) consisting of patients who reported low symptom severity and improved consistently over the seven days; Responders Medium Start (29.6%) reported medium to high symptom severity and showed early improvement; Responders High Start (28.7%) reported high symptom severity and improved to a smaller extent; and Non-Responders (22.2%) reported high symptom levels and did not improve over the seven days. From this sub-sample, 5 individuals were removed from further analyses due to their deliberate self-harm occurring before an expression of suicidal ideation.

Therefore, of individuals who exhibited deliberate self-harm *after* reporting suicidal ideation *(N = 928)*; Non-Responders (14.6%) were significantly more likely to self-injure than Responders High Start (4.9%), Responders Medium Start (1.4%) and Responders Low Start (0.6%). However, there was no significant difference in deliberate self-harm rates between Responders Medium Start and Responders Low Start (see Table 2). Finally, 59.6% of the deliberate self-harm events occurred within 14 days of the first time individuals reported having thoughts about suicide (i.e. day 1 of analyses).

**Table 2:** **Chi-square (** ***χ*** ^**2**^ **) tests for differences in deliberate self-harm rates between groups on the DI-5 Index (N=928)**

**Differences in association with deliberate self-harm**	***χ*** ^***2***^ **value (** ***df*** **)**	**Significance**	**Effect size (** ***Φ*** **)**
DI-5 index overall	52.82 (*3*)	*p<.01*	.24
Non-Responders vs. Responders low start	24.78 (*1*)	*p<.01*	.26
Non-Responders vs. Responders medium start	32.00 (*1*)	*p<.01*	.26
Non-Responders vs. Responders high start	12.84 (*1*)	*p<.01*	.17
Responders high start vs. Responders low start	6.50 (*1*)	*p<.05*	.12
Responders high start vs. Responders medium start	5.67 (*1*)	*p<.05*	.10
Responders low start vs. Responders medium start	.70 (*1*)	p =.40	.04

Since groups of patients sharing early change were found to exist, and they significantly differed in their rates of deliberate self-harm, the next step in the analysis was to check if continuously measuring symptoms provided more predictive power over deliberate self-harm than cross sectional measurements on day 1. Since Non-Responders were at the highest risk of deliberate self-harm, when group membership was regressed on deliberate self-harm as a categorical variable (Non-Responder = 1, other groups = 0), being a Non-Responder significantly increased the odds of deliberate self-harm by an odds ratio of 6.67 (*Exp. B (6.67)*, *p < .01; B* = *1.89*(SE = .31), Nagelkerke *R*^*2*^ *=* .12). Therefore, being grouped as a Non-Responder provided more than six times predictive power over deliberate self-harm in this sample than the cross-sectional measure of initial distress (DI-5 scores on day 1), (*Exp. B (1.2)*, *p < .01;*B = *.15*(SE = .04), Nagelkerke *R*^*2*^ *=* .06).

An example of how results can be organised is provided below in Figure 1. Figure 1 displays the differences in risk between individuals in this population who reported suicidal ideation before exhibiting deliberate self-harm and those who never did during their current admissions.

*Part 3: predicting group membership*

Results suggested that it was important to determine which patients would be grouped as *Non-Responders*; as they were at the highest risk of deliberate self-harm in this sample and would have caused concern for any clinical staff due to high distress levels which did not improve. Therefore, one logistic regression analyses explored whether gender, age and number of previous admissions to hospital could predict which patients would be grouped as Non-Responders. Another logistic regression explored whether diagnoses could predict if individuals would be Non-Responders or not. Analyses revealed that females were more likely to be Non-Responders than males in this sample (*Exp. B* (*2.46*), *p < .01; B* = *.90*(SE = .21), Nagelkerke *R*^*2*^ *= .04*). Further, individuals with personality disorders were significantly more likely to be Non-Responders (*Exp. B* (*4.60*), *p < .01; B* = *1.53*(SE = .30), Nagelkerke *R*^*2*^ *= .04*) where 54.2% (n = 26) of patients with this diagnosis were Non-Responders. Conversely, individuals with substance abuse disorders were significantly less likely to be Non-Responders (*Exp. B* (.27), *p < .05; B* = −*1.3*(SE = .40), Nagelkerke *R*^*2*^ *= .02*) where only 7.8% (n = 7) of patients with this diagnosis were Non-Responders. Age, number of admissions and other diagnoses did not show significant relationships with being grouped as a Non-Responder on the DI-5 Index.

## Discussion

The aims of this study were to determine whether distinct sub-groups of inpatients reporting thoughts about suicide existed based on shared early responses to treatment. It was predicted that some groups would be at a higher risk of deliberate self-harm. It was also expected that when the sum of scores on the DI-5 (suicidal ideation, depression, anxiety, feelings of worthlessness and perceived inability to cope; [27]), was monitored, it would allow for more precision in identifying those at risk of deliberate self-harm than cross-sectional measurements alone (i.e. initial distress measured by DI-5 scores on day 1). Finally, potential predictors of group membership (age, gender, number of admissions to hospital and diagnoses) were explored.

### Daily monitoring, groups and deliberate self-harm

Consistent with previous research [29], it was found that patients in this sample *could* be meaningfully grouped according to their reported improvements in psychological distress during the early stages of treatment. Indeed, these groups acted as a *greater* predictor of deliberate self-harm compared to measures of initial psychological distress. Therefore, continuously monitoring distress improved the precision with which risk of deliberate self-harm could be estimated in this sample.

For example, Non-Responders (individuals who reported severe symptoms and did not improve over seven consecutive days) were significantly more likely to use deliberate self-harm than any other group (see Figures 2 and 1). Further, when group membership was regressed on deliberate self-harm, being a Non-Responder significantly increased the odds of deliberate self-harm by a factor of 6.67 compared to just 1.20 by higher symptom scores on day 1. In addition, Responders High Start and Responders Medium Start (see Figure 2) began with similar distress severity; but it was the magnitude of change between days 1 and 2 (i.e. early change) which appeared to significantly distinguish them in terms of risk of deliberate self-harm. In this way, the use of daily monitoring made it possible to differentiate between those who were significantly more likely to use deliberate self-harm based on their group membership. It is however, important to note that the monitoring and grouping of inpatients would act as adjuncts to existing clinical risk evaluation procedures. For example, if a potentially high risk individual was flagged via existing risk management procedures, and continuous monitoring revealed that they did not report any improvement by day three; according to Figure 2, they would likely be a Non-Responder. More specifically, by identifying a Non-Responder who was at a 14.6% risk of deliberate self-harm (compared to a population risk of 4.3%, see Figure 1) one can predict with 3.4 times more accuracy if that individual will engage in deliberate self-harm. Given the potential link between non-suicidal and suicidal deliberate self-harm [55,56], any improved accuracy in predicting deliberate self-harm may improve our ability to predict and prevent more lethal deliberate self-harm in the future.

However, although non-suicidal and suicidal deliberate self-harm have been found to co-occur [1], some researchers suggest that they are associated with different risk factors [1,57]. For example, among incarcerated women with a history of non-suicidal deliberate self-harm, hopelessness was more strongly associated with the frequency of suicide attempts than that of non-suicidal deliberate self-harm [57]. Further, among adolescents being treated for depression; poor family functioning at entry into the program was associated only with suicide attempts, while being younger, female, having anxiety disorders and hopelessness was associated with only non-suicidal deliberate self-harm [58]. Still, it was also found that while non-suicidal and suicidal deliberate self-harm served different functions, both behaviours were attributed to relieving high levels of negative emotions [59]. Consequently, since both suicidal and non-suicidal deliberate self-harm were studied in this sample, results suggest that a potential shared ‘risk’ is a *lack of early improvement* in psychological distress during treatment.

In summary, higher levels of psychological distress, together with a *lack of early improvement* during treatment appear to place individuals in this population at the highest risk of deliberate self-harm. This is consistent with previous research which found that sub-groups of inpatients changing in reported suicidal ideation at different rates were at different risks of engaging in deliberate self-harm [29], and that early change in distress results in more positive outcomes for individuals undergoing psychological treatment [25,26]. Indeed, identifying sub-groups of individuals who change in distress at different rates led to superior predictions in risk of deliberate self-harm than distress scores on day 1 suggesting that continuously monitoring psychological distress amongst inpatients at this psychiatric hospital provided an innovative and useful avenue for risk prediction, and potentially prevention.

### Predicting group membership

Preliminary analyses showed that females were more likely to be Non-Responders than males in this sample. It was also found that patients with personality disorders were significantly more likely to be Non-Responders. Given that 47 out of 48 individuals with personality disorders had diagnoses of Borderline Personality Disorder (BPD), these findings are consistent with previous research which found high rates of deliberate self-harm in patients diagnosed with BPD [41] and a study which found higher mortality rates, including death by suicide in female vs. male patients diagnosed with personality disorders [60]. Due to the predominance of BPD, the lack of early improvement in distress amongst Non-Responders, continued high reported levels of negative affect and deliberate self-harm may all be related to other symptoms of BPD such as emotion dysregulation and intolerance of negative affect [61,62]. Still, more detailed analyses are required to determine why gender appears to be a significant predictor of risk, and which aspects of personality disorders contribute to deliberate self-harm. For example, it was found that higher levels of ‘confusion about self’ and ‘unstable interpersonal relationships’ were associated with both repeated non-suicidal deliberate self-harm and suicide attempts amongst adolescents displaying traits consistent with BPD [42]. Nevertheless, the significant associations of gender and diagnoses with group membership suggest that females and individuals with diagnoses of personality disorders should be closely monitored for risk of deliberate self-harm during treatment.

Conversely, having substance use disorders made individuals significantly less likely to be Non-Responders, placing them at a lower risk of deliberate self-harm in this sample. This could be due to inpatients not having access to substances in a psychiatric facility, which would then reduce the likelihood of them engaging in impulsive behaviours such as deliberate self-harm while intoxicated. However, studies have also found associations between substance abuse and deliberate self-harm. For example, one study found that not only was substance abuse associated with deliberate self-harm during adolescence, but that deliberate self-harm increased the risk of substance abuse during adulthood [63]. Further, a systematic review found deliberate self-harm and psychological distress to be significant correlates of substance abuse [64]. Perhaps, the lack of association between being Non-Responders and substance abuse in this sample may also be because only primary diagnoses were examined. Substance abuse may have been a comorbid problem in some cases.

Finally, contrary to expectations, age was not a significant predictor of group membership. This could be due to the wide range of ages found in this sample (*M* = 38.95, *SD* = 14.64), including much fewer individuals under 18 years old (8.2%) than over 18; and results of previous research suggesting that deliberate self-harm is more common, and chronic in adolescents experiencing psychological distress than in adults [2,65]. This may also explain the low overall rate of deliberate self-harm (7.1%) in this sample.

Undoubtedly, relationships between diagnoses and group membership need to be explored in more detail before strong conclusions about risk of deliberate self-harm can be drawn. Further study is important, because if information about group predictors can be used to make accurate predictions of individuals at the highest (and lowest) risk of deliberate self-harm, based on their probable group membership; it can help prevent adverse incidents from occurring at all. Future research should explore more predictors of group membership and any interactions between them. For example, dividing risk factors into demographic (e.g. gender), clinical, psychosocial (e.g. social support) and institutional factors (e.g. staff training) may help disentangle predictors of deliberate self-harm and group membership [66]. Finally, studying relationships between theoretical constructs such as perceived burdensomeness, thwarted belongingness and acquired capability for suicide [10], together with diagnoses and demographic factors; and their effects on group membership may provide characteristics which place individuals at higher risks of deliberate self-harm.

### Limitations

Firstly, the selection of participants in this study may have resulted in a sample consisting of more severe patients (inpatients for a minimum of seven days, and consisting only of people who reported suicidal ideation). Further, the level of missing data on certain days may limit the generalizability of risk values found in this sample. Missing data on some days may have been due to procedural reasons (e.g. newly admitted patients, or soon to be discharged) or a number of other factors such as a lack of opportunity to complete the measure due to missing a treatment session. Therefore, the numbers in Figure 1 regarding the risk of deliberate self-harm should be interpreted with caution. Rather than absolute risk values which can be generalised to all inpatient populations, they should be seen as the relative difference in risk of deliberate self-harm in this sample. Nevertheless, the process of determining group membership and resulting risk of deliberate self-harm through continuous measurement can still be applied to other populations.

Secondly, since the number of deliberate self-harm events recorded in this sample was based only on those reported by hospital staff; there may have been incidents which staff were not aware of, and this might explain the low reported rate of deliberate self-harm in this population.

Furthermore, it was found that five individuals who exhibited deliberate self-harm did so before reporting suicidal ideation. This could be because these incidents did not indicate an acquired capacity for future suicidal behaviour, or that suicidal ideation developed as a result of the deliberate self-harm. Nonetheless, since this model aimed to predict deliberate self-harm based on changes in psychological distress after self-reported suicidal ideation; predicting deliberate self-harm which did not follow reported thoughts about suicide are beyond the scope of this study.

Finally, it was found that the two individuals who attempted suicide (self-reported) were grouped as Non-Responder and Responder High Start. Therefore, measuring non-suicidal deliberate self-harm and suicide attempts separately; and how they may be distributed in groups provides an avenue for future research with larger samples. Indeed, separating non-suicidal and suicidal deliberate self-harm could help clarify both the link and the differences between risk factors for the two behaviours. This separation may also clarify relationships between any predictors of group membership, and future research should take this into account.

## Conclusions

Results suggest that amongst inpatients reporting suicidal ideation; the daily monitoring of their indicators of psychological distress allowed them to be meaningfully grouped according to shared early change during treatment. This grouping allowed significantly more precision in predicting risk of deliberate self-harm according to group membership compared to cross-sectional measures alone. For example, the group with high initial distress and no early change was at the highest risk of deliberate self-harm. Results also suggest that females and those with diagnoses of personality disorders should be closely monitored for risk of deliberate self-harm. These findings present a novel and practical approach for the first steps in mitigating the risk of deliberate self-harm in clinical populations.
